# Gut microbe *Terrisporobacter* promotes papillary thyroid carcinoma progression by upregulating the *NTRK1* oncogene and fostering an immunosuppressive tumor microenvironment

**DOI:** 10.3389/fimmu.2026.1740257

**Published:** 2026-03-25

**Authors:** Jia Li, Jie Shen, Dongyan Lu, Enci Ding, Lijuan Wei

**Affiliations:** Department of Nuclear Medicine, Tianjin First Central Hospital, Tianjin, China

**Keywords:** gut microbiota, immunosuppression, M2 macrophages, Mendelian randomization, metabolites, NTRK1, papillary thyroid carcinoma, *Terrisporobacter*

## Abstract

Growing evidence suggests a link between the gut microbiome and papillary thyroid carcinoma (PTC), but the causal relationships and the impact on the tumor immune microenvironment (TME) are poorly understood. This study aimed to elucidate the causal role of specific gut microbes in PTC and uncover the underlying immunological and molecular mechanisms. We employed a multi-stage design, beginning with a two-sample Mendelian randomization (MR) analysis using large-scale GWAS data to infer causality. Findings were then validated in 450 PTC patients from The Cancer Genome Atlas (TCGA) by analyzing correlations between microbial abundance, gene expression, immune cell infiltration, and survival. Finally, the core mechanism was confirmed through extensive *in vitro* experiments with PTC cell lines. Our MR analysis identified a causal association between a genetically predicted higher abundance of the genus *Terrisporobacter* and an increased risk of PTC (Odds Ratio [OR] = 2.06, 95% Confidence Interval [CI]: 1.34-3.16). In the TCGA cohort, higher intratumoral signals of *Terrisporobacter* was significantly correlated with an immunosuppressive TME, characterized by increased infiltration of M2 macrophages (ρ = 0.25, p < 0.001) and decreased CD8+ T cells (ρ = -0.19, p = 0.008). Mechanistically, *Terrisporobacter* abundance was also strongly associated with the upregulation of the oncogene *NTRK1* (ρ = 0.35, p < 0.001), which independently predicted poorer overall survival (Hazard Ratio [HR] = 2.15, p = 0.004). *In vitro* experiments confirmed that supernatant from *Terrisporobacter* culture not only upregulated *NTRK1* expression and promoted PTC cell proliferation but also enhanced invasion and induced cell de-differentiation. Importantly, pharmacological inhibition of TRK signaling reversed the bacteria-induced aggressive phenotype. Our integrated analysis provides robust, multi-layered evidence for a causal role of *Terrisporobacter* in promoting PTC progression. We define a novel gut-thyroid axis where *Terrisporobacter* contributes to PTC development by upregulating the *NTRK1* oncogene and shaping a pro-tumorigenic, immunosuppressive microenvironment. These findings reveal a new dimension of host-microbe interaction in thyroid cancer and highlight the TME as a key downstream target of microbial influence.

## 1 Introduction

Thyroid cancer represents 5.11% of all malignant tumors in the head and neck, making it one of the most prevalent types of these malignancies (1). Thyroid nodules are hard and fixed, accompanied by cervical lymphadenopathy, and those with compressive symptoms or thyroid nodules existing for many years suddenly enlarging in a short period should be considered for thyroid cancer. Differentiated carcinomas originating from follicular epithelial cells can be divided into follicular carcinoma (which accounts for 14% of all thyroid cancers) and papillary carcinoma (which accounts for 80% of all thyroid cancers) (1). Medullary carcinoma originating from C cells accounts for 4%, while the remaining 2% are highly invasive anaplastic carcinomas (2). Statistical studies on the incidence of thyroid cancer have shown that the incidence of thyroid cancer worldwide has been increasing year by year (3). In 2023, nearly 43,720 new cases were reported in the United States, ranking first among endocrine malignancies, with approximately 655 deaths attributed to thyroid cancer each year (4). Papillary thyroid carcinoma (PTC) may spread more to cervical lymph nodes and less to the lungs. With early diagnosis, PTC can be curable (5).

Currently, the only confirmed environmental risk factor is ionizing radiation, especially exposure during childhood (6). Additionally, a meta-analysis found that overweight and obesity are both risk factors for thyroid cancer, with relative risks (RRs) of 1.13 (95% CI = 1.04-1.22) and 1.29 (95% CI = 1.18-1.41), respectively (7). Franchini et al. (8) suggested that overweight or obesity leading to thyroid cancer is associated with thyroid hormones, insulin resistance, adipokines, inflammation, and sex hormones.

A key component in determining health and illness is the microbiome. Microbial ecology theory suggests that the ecological balance among the nervous system, endocrine system, microbiota, metabolism, and immune system is a significant characteristic of life sustainability (9). The oral and intestinal tracts are two important gastrointestinal structures, forming a microbial consortium primarily responsible for metabolic processes and energy intake, crucial for human health. The gut microbiota comprises nearly 1200 bacterial species, as well as archaea, fungi, and viruses. Major bacteria include Actinobacteria, Firmicutes and Bacteroides. Immunity, hormone balance, metabolic balance, and digestive balance all depend on these microorganisms. Dysbiosis, which is characterized by disruption of the makeup of the gut microbiota, results in an imbalance in the microbial ecology and a decrease in microbial diversity. Alterations in the makeup of the gut microbiota are mostly linked to metabolic and inflammatory illnesses, including diabetes, autoimmune diseases, and inflammatory bowel diseases (9). Crucially, obesity and overweight status—known risk factors for thyroid cancer—are strongly associated with significant alterations in the gut microbiota composition, often characterized by a reduction in microbial diversity and a shift in the Firmicutes/Bacteroidetes ratio (10). The makeup of the gut microbiota becomes more complicated as one ages. Furthermore, long-term dietary modifications or drug use may also significantly affect an adult’s gut microbiome composition (11). Numerous studies have shown the connection between thyroid function and gut flora (12). The interaction between triiodothyronine (T3) and the major thyroid hormone receptor alpha-1 (TRα-1) in the colon regulates the equilibrium of intestinal epithelium. This internal equilibrium depends on the tight control of the local T3 concentration, which is provided by intraluminal deiodinases and particular TH transporters. The immune and endocrine systems depend on a healthy gut microbiota and metabolite balance (12, 13). The gut microbiota influences the immune system through several metabolites, which also participate in regulating thyroid function (12).

Despite the fact that observational studies have linked the risk of acquiring PTC to the gut microbiota and its metabolites, the underlying causal mechanisms of these associations remain poorly elucidated. Recent investigations have attempted to elucidate the causal relationships between gut microbiota, their metabolites, and cancer using Mendelian randomization (MR) analysis (14, 15). Using observational data, MR analysis uses an instrumental variable (IV) technique to establish causal links between exposures and outcomes (16).

In this study, we utilized MR analysis to ascertain the causal links among PTC, metabolites and gut microbiota. Furthermore, to bridge the gap between genetic prediction and clinical reality, we performed a validation study using data from The Cancer Genome Atlas (TCGA) to explore the associations of MR-identified microbial taxa with key gene expression and clinical outcomes in PTC patients. To provide direct evidence for our key finding, we further performed *in vitro* experiments to validate the regulatory effect of a risk-associated microbe on its target oncogene and on PTC cell behavior, specifically examining proliferation, invasion, and de-differentiation. Through our integrated analysis, we identified several genetic variants associated with alterations in bacterial composition, potentially influencing the pathogenesis of PTC. Our findings lay the groundwork for further research aimed at the diagnosis and treatment of PTC.

## 2 Methods

### 2.1 Study design

**Figure 1:**
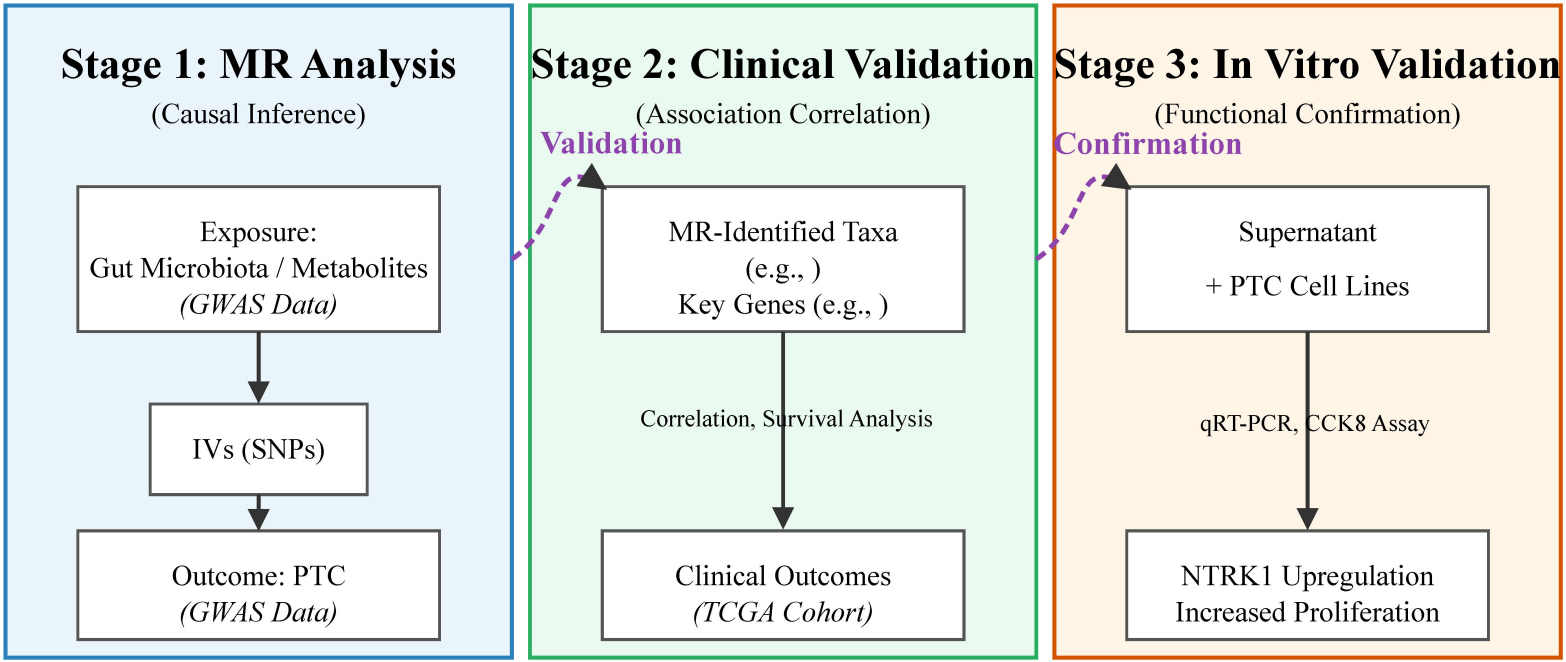
Schematic diagram of the study design. The study comprises three integrated stages: **(A)** Mendelian randomization (MR) analysis to infer causality between exposures (gut microbiota/metabolites) and outcome (PTC). (Gut microbiota GWAS n=18,340; PTC GWAS n=392,423). **(B)** Clinical and bioinformatic validation in the TCGA cohort to assess correlations between microbial signatures, key genes, immune microenvironment, and clinical outcomes. (TCGA Cohort n=450). **(C)** *In vitro* experimental validation to confirm the biological effect of the identified risk microbe on PTC cells. Note: The “Upregulation” in Stage 3 refers specifically to *NTRK1* Upregulation.

Utilizing genome-wide association study (GWAS) summary data, we conducted a two-sample Mendelian randomization (MR) analysis to elucidate the causal effects of gut microbiota and metabolites on PTC. The MR design necessitates three key prerequisites (refer to **Figure 1**): (1) validation that the selected genetic variants (used as instrumental variables, IVs) are robustly associated with the exposure (metabolites and gut microbiota); (2) assurance that the genetic instruments are not associated with any confounding variables; and (3) confirmation that the genetic variants are exclusively linked to PTC via pathways involving metabolites and gut microbiota, ruling out any other pleiotropic impacts. Following the MR analysis, we designed a clinical validation study using an independent public cohort to corroborate our primary findings and conducted subsequent *in vitro* experiments for mechanistic validation (**Figure 1**). Ethical approval was waived by the ethics committee of our hospital because this study exclusively used publicly available, anonymized summary-level data and patient data from the TCGA database, with no direct involvement of human participants or animals.

### 2.2 Data sources

The data used included gut microbiota, metabolites and PTC. The data of gut microbiota were sourced from the MiBioGen consortium (https://mibiogen.gcc.rug.nl/) involving 18,340 cases in 2021; and the data of metabolites were from a GWAS including 7,824 participants reported in 2023 (https://www.frontiersin.org/journals/microbiology/articles/10.3389/fmicb.2023.1087622/full) (17), respectively. Additionally, the summary statistics for PTC were obtained from FinnGen (https://r9.risteys.finngen.fi/endpoints/C3_THYROID_PAPILLARY_ADENO) with the sample size of 392,423 published in 2022. All participants in these GWAS were of European ancestry, which minimizes bias from population stratification.

### 2.3 Selection of instrumental variables

To ensure the validity of IVs, we applied a stringent selection process. First, single-nucleotide polymorphisms (SNPs) significantly associated with each exposure (gut microbiota taxa and metabolites) were selected using a genome-wide significance threshold of p < 5 × 10^-8^. To obtain a sufficient number of IVs for robust analysis, we relaxed this to a locus-wide significance threshold of p < 1 × 10^-5^ for exposures with few genome-wide significant SNPs, a common practice in MR studies of the microbiome (18). Second, to ensure independence among IVs, we performed linkage disequilibrium (LD) clumping with an r² threshold of 0.001 and a clumping window of 10,000 kb, using the 1000 Genomes European reference panel. Third, to assess the strength of the selected IVs, we calculated the F-statistic for each SNP using the formula F = (Beta/SE)² and ensured that the mean F-statistic for each exposure was well above the conventional threshold of 10 to mitigate weak instrument bias (19).

### 2.4 Mendelian randomization analysis

Upon selecting eligible IVs, we proceeded with MR analysis to ascertain the causal association between gut microbiota, metabolites, and PTC risk. To evaluate the causal influence, a number of analytical techniques were used, such as MR-Egger, inverse-variance weighted (IVW), simple and weighted modes, and weighted median. The other four approaches were used as a complement to the main analytical strategy, which was the IVW method. The IVW method provides the most precise estimate when all IVs are valid. Results were presented as odds ratios (ORs) with 95% confidence intervals (CIs). Due to the limited sample size of some exposures, and this study’s exploratory nature, a p-value < 0.05 was considered suggestive of a causal association without strict multiple testing correction. Power calculations were carried out using the mRnd website.

### 2.5 Sensitivity analysis

We conducted several sensitivity analyses to assess the robustness of our findings. Horizontal pleiotropy was evaluated for significant estimates using the intercept term derived from MR-Egger regression (19). MR-PRESSO (Pleiotropy RESidual Sum and Outlier) was used to investigate the existence of pleiotropic biases, and outliers were eliminated to account for pleiotropic effects. Using Cochran’s Q statistics, statistical heterogeneity in the IVW meta-analysis was evaluated. Finally, a “leave-one-out” analysis was performed to determine if any single SNP was driving the causal estimate. All analyses were conducted using the TwoSampleMR (v0.5.6) and MR-PRESSO packages in R (version 4.2.1).

### 2.6 Clinical validation in the TCGA cohort

**Figure 2:**
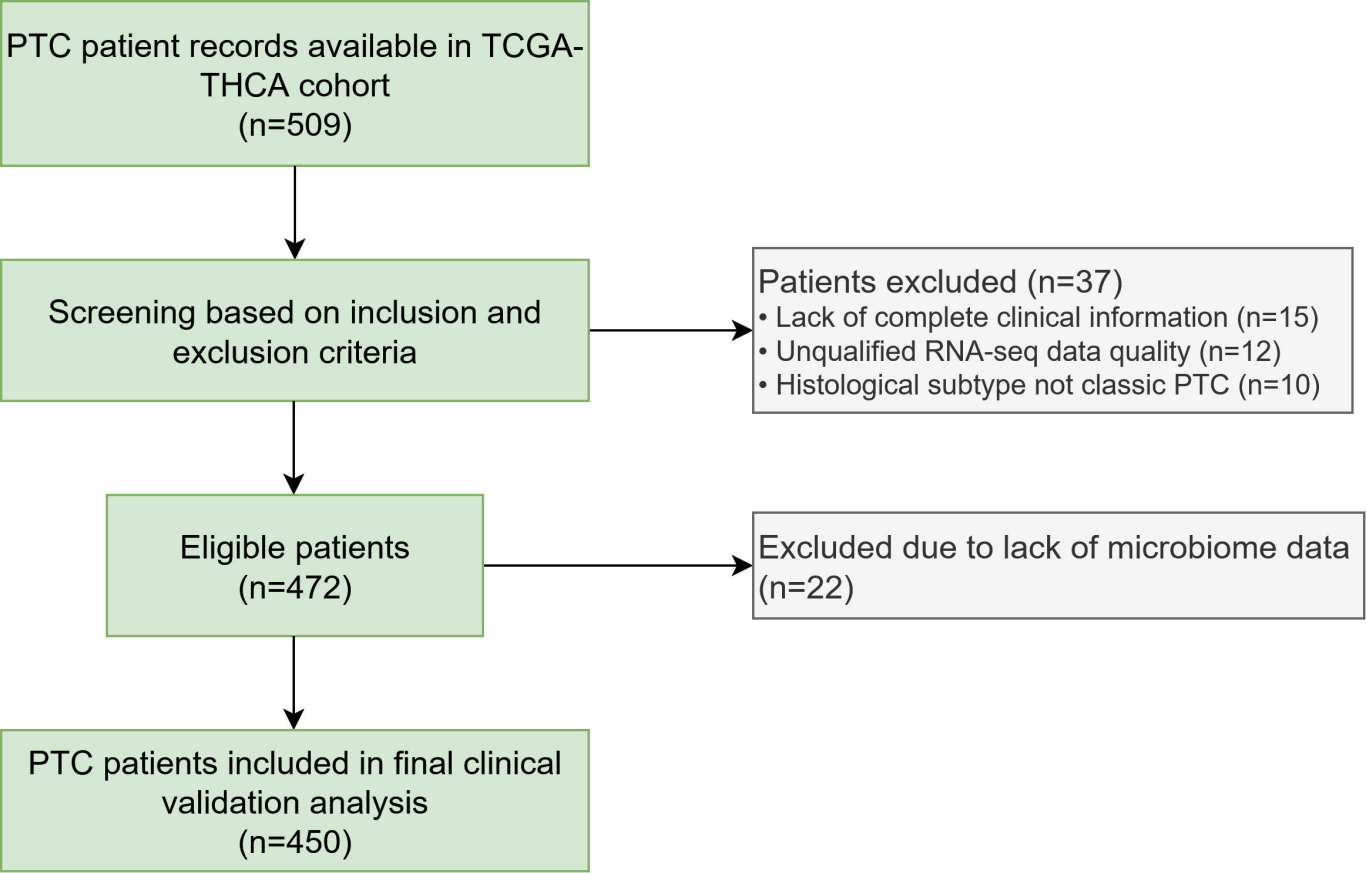
Flowchart of patient selection from The Cancer Genome Atlas (TCGA) cohort. A total of 509 records from the TCGA-THCA project were initially screened. After applying inclusion and exclusion criteria, 450 patients with papillary thyroid carcinoma (PTC) were included in the final analysis for clinical validation.

To validate our MR findings, we utilized data from The Cancer Genome Atlas Thyroid Carcinoma (TCGA-THCA) project, accessed via the GDC Data Portal. We included patients with a confirmed diagnosis of PTC, available RNA sequencing (RNA-seq) data, and complete clinical follow-up information. A patient selection flowchart is provided in **Figure 2**. Inclusion criteria were: (1) pathologically confirmed papillary thyroid carcinoma; (2) available RNA-seq data; and (3) complete clinical follow-up information. Exclusion criteria included: (1) history of other malignancies; (2) incomplete clinical data; and (3) non-PTC histological subtypes. The final cohort consisted of 450 PTC patients. We used the bioinformatics pipeline PathSeq to quantify the relative abundance of microbial reads, including the MR-identified genera, from the raw RNA-seq data. Gene expression was quantified as Transcripts Per Million (TPM). We focused on key genes identified in our functional enrichment analysis (*GLS2, NTRK1, PDE4D, RYR2*). To address the potential sex bias in the cohort, sex-stratified correlation analyses were performed.

Statistical analyses for the clinical validation included: (1) Spearman’s rank correlation to assess the association between the relative abundance of MR-identified bacteria and the expression of key genes. (2) The Wilcoxon rank-sum test to compare gene expression or microbial abundance between subgroups (e.g., tumor vs. adjacent normal tissues, early vs. advanced clinical stages). (3) Kaplan-Meier analysis with the log-rank test to evaluate the association between high and low expression/abundance groups (stratified by the median) and overall survival (OS). (4) A multivariable Cox proportional hazards model, adjusting for age, gender, and tumor stage, was used to calculate hazard ratios (HRs) and 95% CIs. All clinical data analyses were performed in R, with a two-sided p-value < 0.05 considered statistically significant.

### 2.7 Tumor immune microenvironment analysis

To investigate the potential immunomodulatory role of the identified microbiota, we analyzed the tumor immune microenvironment of the 450 PTC patients in the TCGA cohort. The relative abundance of 22 types of tumor-infiltrating immune cells was quantified from the TPM-normalized RNA-seq data using the CIBERSORTx algorithm. We then assessed the correlation between the abundance of *Terrisporobacter* and the infiltration levels of key immune cells, such as M2 macrophages and CD8+ T cells, using Spearman’s rank correlation. Differences in immune cell infiltration between high and low *Terrisporobacter* abundance groups (stratified by the median) were evaluated using the Wilcoxon rank-sum test.

### 2.8 *In vitro* experimental validation

Cell culture and treatment: The human PTC cell line TPC-1 was cultured in RPMI-1640 medium supplemented with 10% fetal bovine serum. *Terrisporobacter glycolicus* (DSM 10793) was cultured anaerobically in modified Schaedler broth at 37 °C in an anaerobic chamber (Coy Laboratory Products) with an atmosphere of 85% N2, 10% H2, and 5% CO2 for 48 hours to reach the stationary phase. Bacterial culture supernatant was collected, filter-sterilized (0.22 μm), and used to treat TPC-1 cells at a 10% (v/v) concentration for 48 hours. Control cells were treated with sterile culture medium.

Quantitative Real-Time PCR (qRT-PCR): Total RNA was extracted from treated and control cells using TRIzol reagent. After reverse transcription, qRT-PCR was performed to measure the relative mRNA expression of *NTRK1* and thyroid differentiation markers (Thyroglobulin [Tg], Sodium Iodide Symporter [NIS], and Thyroid Peroxidase [TPO]). GAPDH was used as the internal control. The 2^-ΔΔCt^ method was used for quantification (20).

Cell Proliferation Assay: TPC-1 cells were seeded in 96-well plates and treated with *Terrisporobacter* supernatant or control medium. Cell proliferation was assessed at 72 hours using the Cell Counting Kit-8 (CCK8) assay according to the manufacturer’s instructions. Absorbance at 450 nm was measured. For the rescue experiment, cells were treated with the supernatant in the presence or absence of the TRK inhibitor Larotrectinib (100 nM).

Transwell Invasion Assay: Cell invasion was assessed using 24-well transwell chambers (8 μm pore size, Corning) pre-coated with Matrigel (BD Biosciences, diluted 1:8). TPC-1 cells (5 × 10^4^) in serum-free medium containing 10% bacterial supernatant or control medium were plated in the upper chamber. Medium with 20% FBS was added to the lower chamber. After 24 hours, invaded cells were fixed with methanol, stained with 0.1% crystal violet, and counted under a microscope in three randomly selected fields per well.

### 2.9 Enrichment analysis

For the gut microbiota and metabolites selected after threshold screening, combined with papillary thyroid carcinoma (PTC), gene matching was performed using the SNPnexus database (https://www.snp-nexus.org/v4/) (21). For these genes, Gene Ontology (GO) and Kyoto Encyclopedia of Genes and Genomes (KEGG) pathway enrichment analyses were conducted using the R package clusterProfiler (p < 0.05).

## 3 Results

### 3.1 Causal effects of gut microbiota on PTC

**Figure 3:**
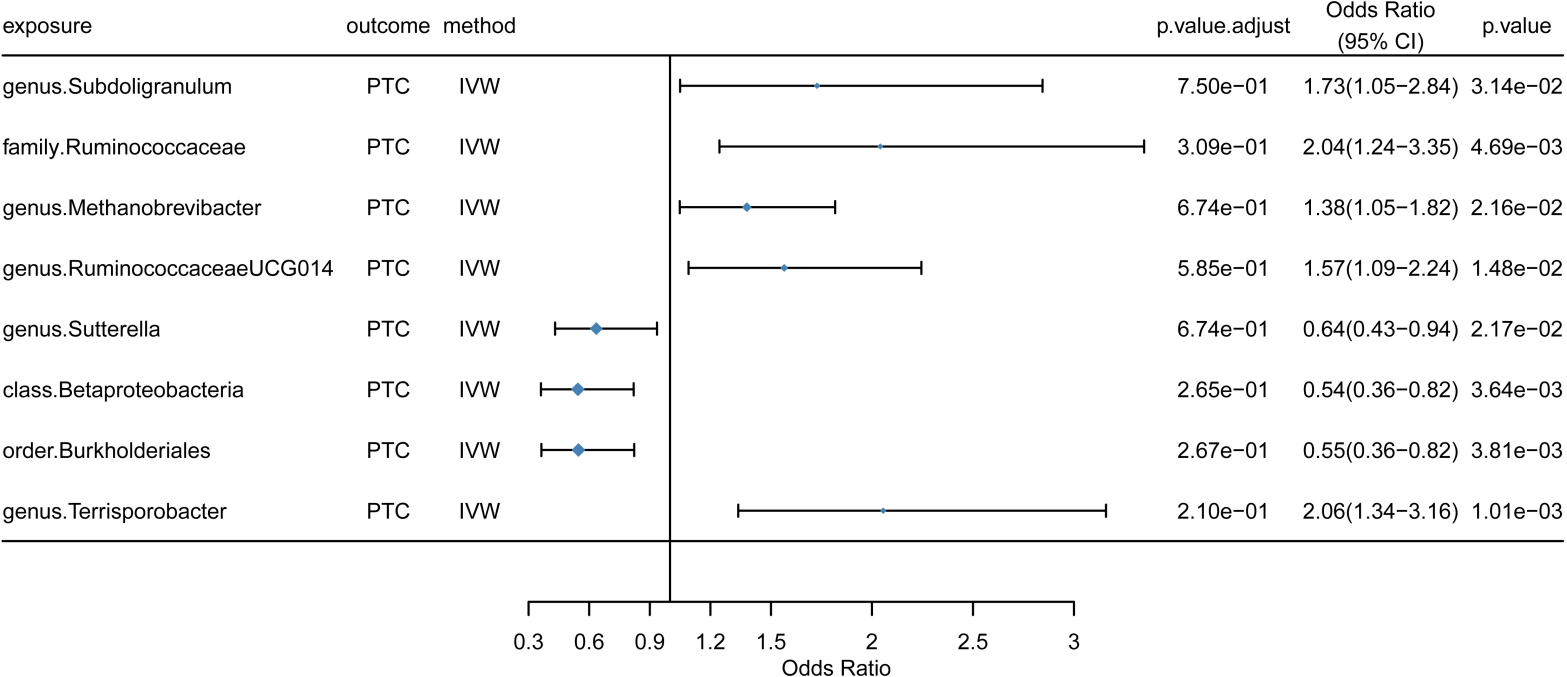
Forest plot of the causal effects of gut microbiota on PTC risk. The plot displays the odds ratio (OR) and 95% confidence interval (CI) for each bacterial taxon found to have a significant causal association with papillary thyroid carcinoma (PTC) in the primary inverse-variance weighted (IVW) analysis. (Gut microbiota GWAS n=18,340; PTC GWAS n=392,423). ORs are estimated per standard deviation increase in the abundance of each taxon. Squares represent the point estimate of the OR, and the horizontal lines represent the 95% CI.

**Figure 4:**
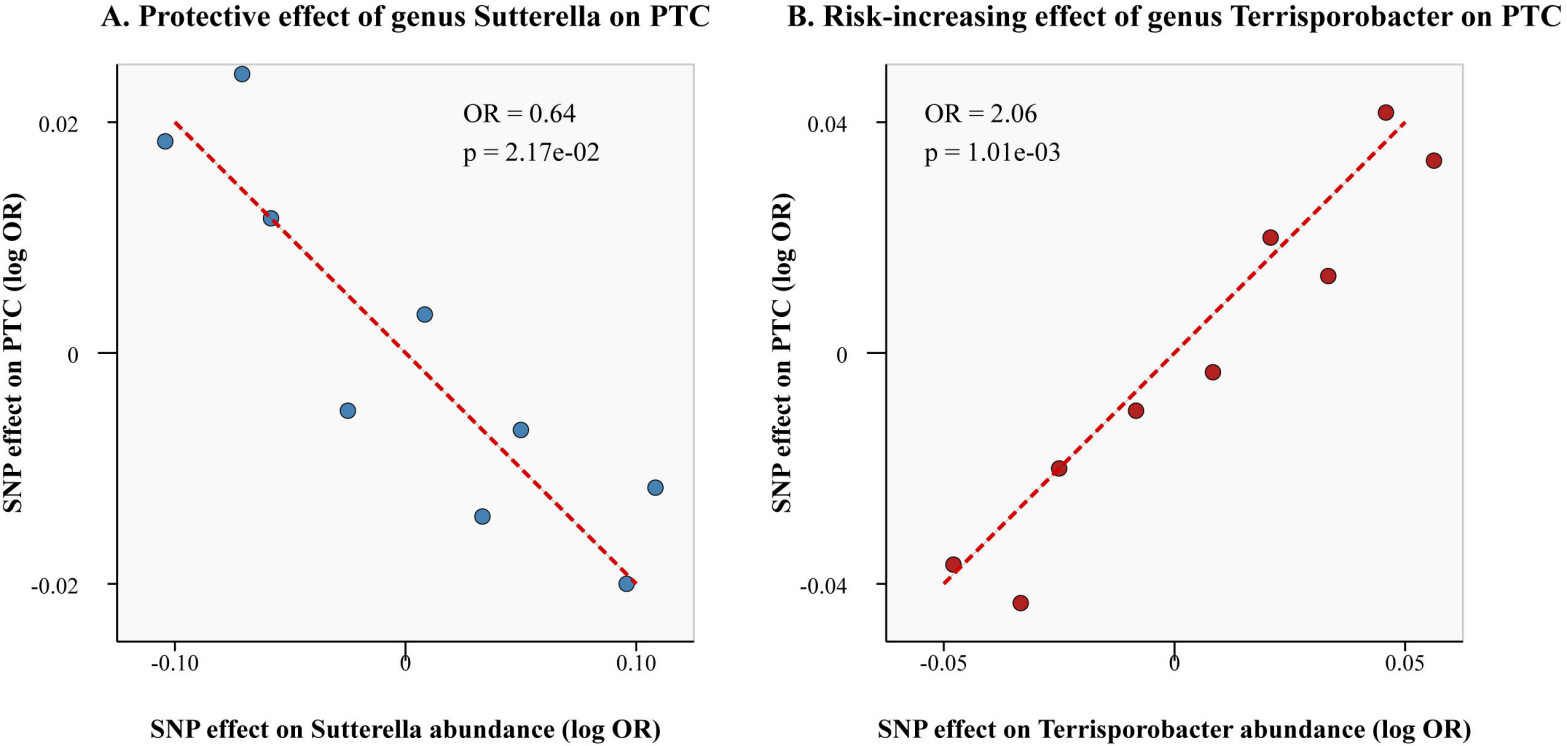
Scatter plots showing the causal effects of representative gut microbiota on PTC. Each point represents a single SNP. The slope of the regression line represents the causal estimate. (Gut microbiota GWAS n=18,340; PTC GWAS n=392,423). **(A)** Protective effect of genus *Sutterella* on PTC. **(B)** Risk-increasing effect of genus *Terrisporobacter* on PTC.

The substantial IVW findings of the gut microbiota’s causal effects on PTC (p < 0.05) are shown in **Figure 3**. At the order level, higher genetically predicted abundance of Burkholderiales was linked to decreased PCT risk (odds ratio [OR] = 0.55, 95% confidence interval [CI]: 0.36-0.82, p = 3.81e-03). A higher genetically predicted abundance of class Betaproteobacteria (OR = 0.54, 95% CI: 0.36-0.82, p = 3.64e-03) was linked to a decreased incidence of PCT at the class level. At the genus level, a higher genetically predicted abundance of Sutterella (OR = 0.64, 95% CI: 0.43-0.94, p = 2.17e-02) was linked to a decreased risk of PCT, suggesting a protective effect. However, family Ruminococcaceae (OR = 2.04, 95% CI: 1.24-3.35, p = 4.69e-03) was linked to a greater incidence of PCT at the family level. Furthermore, at the genus level, a positive correlation was found between increased PTC risk and Subdoligranulum (OR = 1.73, 95% CI: 1.05-2.84, p = 3.14e-03), Methanobrevibacter (OR = 1.38, 95% CI:1.05-1.82, p = 2.16e-02), RuminococcaceaeUCG014 (OR = 1.57, 95% CI: 1.09-2.24, p = 1.48e-02), and Terrisporobacter (OR = 2.06, 95% CI: 1.34-3.16, p = 1.01e-03). Certain relationship findings were supported using the weighted median technique. Scatter plots visually represent the causal association between representative gut microbiota (the most significant protective and risk factors) and PTC (**Figure 4**). Detailed information regarding the number of SNPs and their rsIDs for each significant exposure is provided in **Supplementary Table 1**.

### 3.2 Causal effects of metabolites on PTC

**Figure 5:**
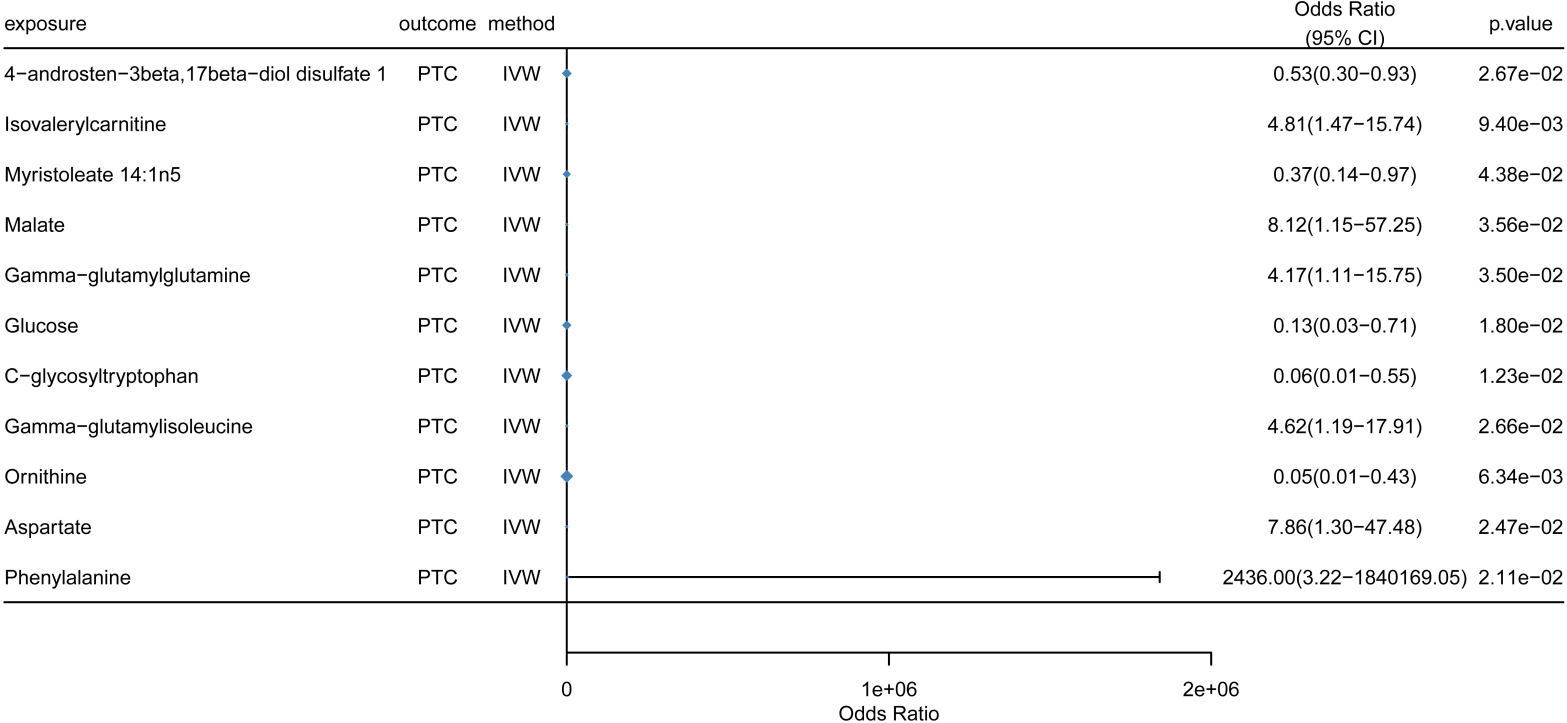
Forest plot of the causal effects of metabolites on PTC risk. The plot displays the odds ratio (OR) and 95% confidence interval (CI) for each metabolite with a significant causal association with papillary thyroid carcinoma (PTC). (Metabolite GWAS n=7,824; PTC GWAS n=392,423). ORs are estimated per standard deviation increase in metabolite concentration.

**Figure 5** presents the significant IVW results of causal effects of metabolites on PTC (p < 0.05). Genetically predicted higher concentrations of Isovalerylcarnitine (OR = 4.81, 95%CI: 1.47–15.74, p = 9.40e-03), Malate (OR = 8.12, 95%CI: 1.15–57.25, p=3.56e-02), Gamma-glutamylglutamine (OR = 4.17, 95%CI: 1.11–15.75, p =3.51e-02), Gamma-glutamylisoleucine (OR = 4.62, 95%CI: 1.19–17.91, p=2.68e-02), Aspartate (OR = 7.86, 95%CI: 1.30–47.48, p=2.47e-02), and Phenylalanine (OR = 2436.00, 95%CI: 3.22-1.84e+06, p=2.11e-02) were associated with higher PTC risk. However, as shown in **Figure 5**, 4-androsten-3beta,17beta-diol disulfate 1 (OR = 0.53, 95%CI: 0.30–0.93, p = 2.67e-02), Myristoleate (14:1n5) (OR = 0.37, 95%CI: 0.14–0.97, p=4.38e-02), Glucose (OR = 0.13, 95%CI: 0.03–0.71, p=1.80e-02), C-glycosyltryptophan (OR = 0.06, 95%CI: 0.01–0.55, p=1.23e-02) and Ornithine (OR = 0.05, 95%CI: 0.01–0.43, p=6.34e-03) were identified as protective factors against PTC.

### 3.3 Sensitivity analyses

Since all of the chosen IVs’ F-statistics were more than 10, there was no weak IV bias. The MR-Egger intercept’s p-value was greater than 0.05 for all significant associations, which suggests that neither directional horizontal pleiotropy nor possible outlier IVs were present. The Cochran’s Q test showed no significant heterogeneity for the main findings (p > 0.05). Furthermore, the results of the leave-one-out test supported the robustness of our findings by indicating that no one SNP significantly affected the MR estimate.

### 3.4 Clinical validation of key microbial signatures and genes in the TCGA cohort

To clinically validate our MR findings, we analyzed data from 450 PTC patients from the TCGA-THCA cohort. The patient selection process is detailed in **Figure 2**, and the baseline clinicopathological characteristics of the cohort are summarized in **Table 1**. The cohort was predominantly female (72.9%) with a median age of 46 years, and the majority of tumors were classified as Stage I (55.1%).

**Table 1:** Baseline clinicopathological characteristics of the TCGA-THCA cohort (n=450).

Characteristic	Value
Age (years), Median [IQR]	46 [35-59]
Gender, n (%)	
Male	122 (27.1)
Female	328 (72.9)
Pathological Stage, n (%)	
Stage I	248 (55.1)
Stage II	59 (13.1)
Stage III	95 (21.1)
Stage IV	48 (10.7)
Tumor Status at last follow-up, n (%)	
Tumor Free	401 (89.1)
With Tumor	49 (10.9)
Vital Status, n (%)	
Alive	425 (94.4)
Deceased	25 (5.6)

Data are presented as median [interquartile range, IQR] or number (%).

**Figure 6:**
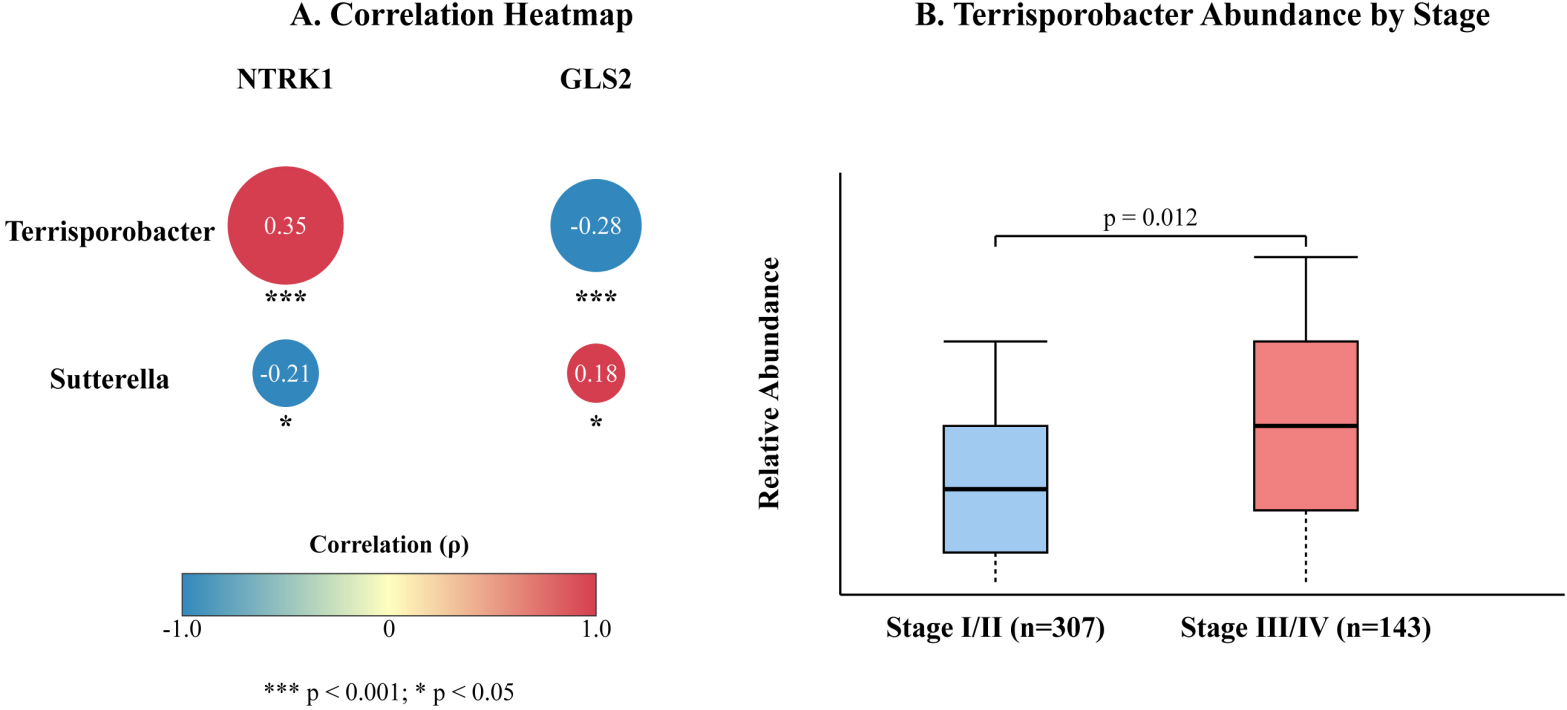
Clinical validation of microbial and genetic markers in the TCGA-THCA cohort (n=450). **(A)** Correlation heatmap showing Spearman’s correlation coefficients (ρ) between the relative abundance of MR-identified bacteria (*Terrisporobacter, Sutterella*) and the expression of key genes (*NTRK1, GLS2*). Color intensity and size of circles indicate the strength and direction of the correlation. Significance levels are denoted by asterisks: ***p < 0.001; *p < 0.05. **(B)** Box plot comparing the relative abundance of genus *Terrisporobacter* between patients with early-stage (I/II, n=307) and advanced-stage (III/IV, n=143) PTC. The p-value was calculated using the Wilcoxon rank-sum test. The central line in the box plot represents the median, the box limits represent the interquartile range (IQR), and the whiskers extend to 1.5 times the IQR.

We first investigated the link between the MR-identified microbes and key genes from our pathway analysis. We focused on Terrisporobacter (a PTC risk factor) and Sutterella (a protective factor). A correlation analysis revealed a significant positive correlation between the relative abundance of Terrisporobacter and the mRNA expression of the oncogene NTRK1 (Spearman’s ρ = 0.35, p < 0.001), while it was negatively correlated with the tumor suppressor gene GLS2 (ρ = -0.28, p < 0.001). Conversely, the abundance of Sutterella showed a negative correlation with NTRK1 expression (ρ = -0.21, p = 0.005) (**Figure 6A**). Furthermore, we observed that the abundance of Terrisporobacter was significantly higher in patients with advanced pathological stages (Stage III/IV) compared to early stages (Stage I/II) (p = 0.012, **Figure 6B**).

Given the female predominance in PTC, we performed a sex-stratified analysis (**Supplementary Table 2**). The positive correlation between Terrisporobacter abundance and NTRK1 expression remained significant in both female (ρ = 0.36, p < 0.001) and male (ρ = 0.31, p = 0.002) subgroups, suggesting the mechanism is not sex-specific. However, the survival disadvantage associated with high NTRK1 was statistically significant in females (p=0.005) but only showed a trend in males (p=0.08), likely due to the smaller sample size of the male cohort.

**Figure 7:**
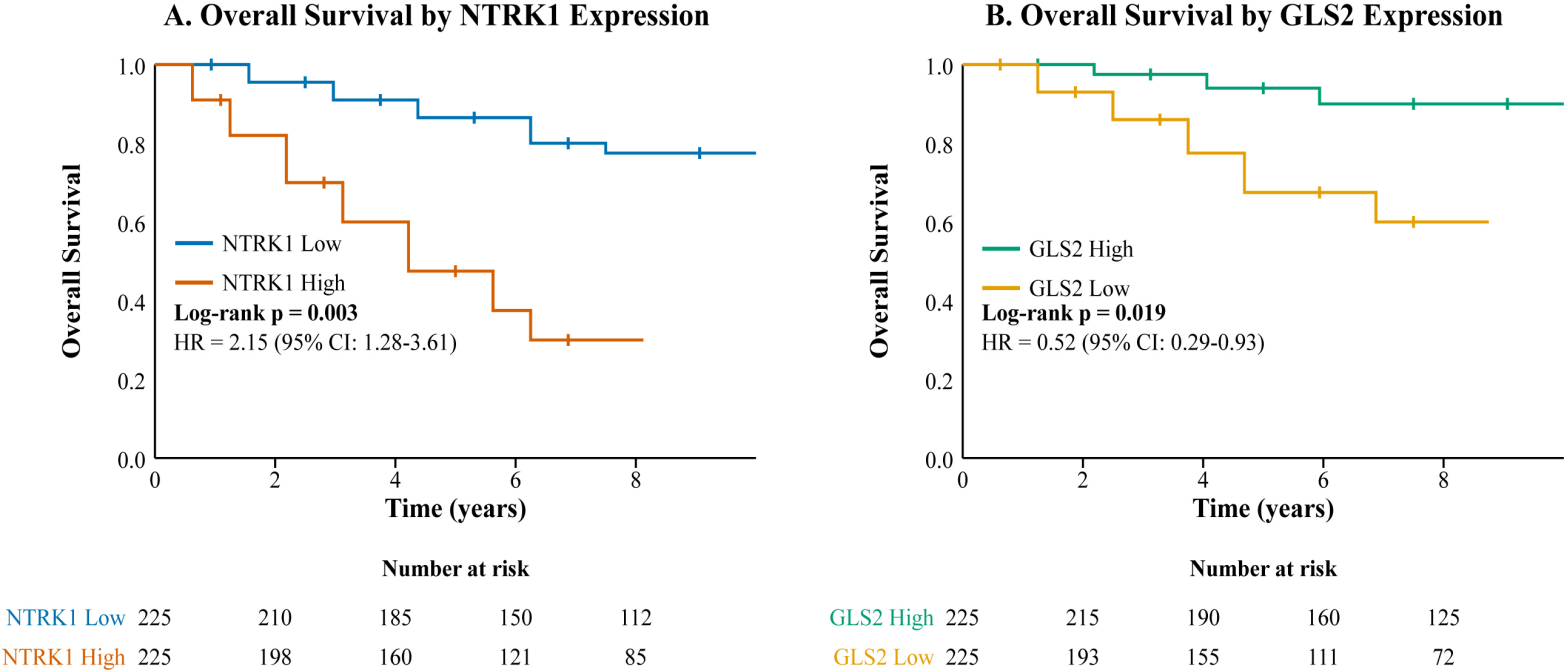
Kaplan-Meier survival analysis for overall survival (OS) in the TCGA-THCA PTC cohort (n=450). Patients were stratified into high and low expression groups based on the median expression value. **(A)** OS curves for patients stratified by *NTRK1* expression (n=450). **(B)** OS curves for patients stratified by *GLS2* expression (n=450). Shaded areas represent the 95% confidence intervals for the survival curves. The log-rank test p-value, hazard ratio (HR), and 95% CI from the multivariable Cox regression model (adjusted for age, gender, and stage) are shown. The table below each plot indicates the number of patients at risk at different time points for each group.

Next, we assessed the prognostic value of these key markers. Kaplan-Meier survival analysis demonstrated that patients with high NTRK1 expression had significantly poorer overall survival (OS) compared to those with low NTRK1 expression s(log-rank p = 0.003) (**Figure 7A**). In a multivariable Cox regression model adjusted for age, gender, and stage, high NTRK1 expression remained an independent predictor of worse OS (HR = 2.15, 95% CI: 1.28-3.61, p = 0.004). Conversely, high expression of the tumor suppressor gene GLS2 was associated with better OS (log-rank p = 0.019; adjusted HR = 0.52, 95% CI: 0.29-0.93, p = 0.027) (**Figure 7B**).

### 3.5 Correlation of *Terrisporobacter* with the tumor immune microenvironment

**Figure 8:**
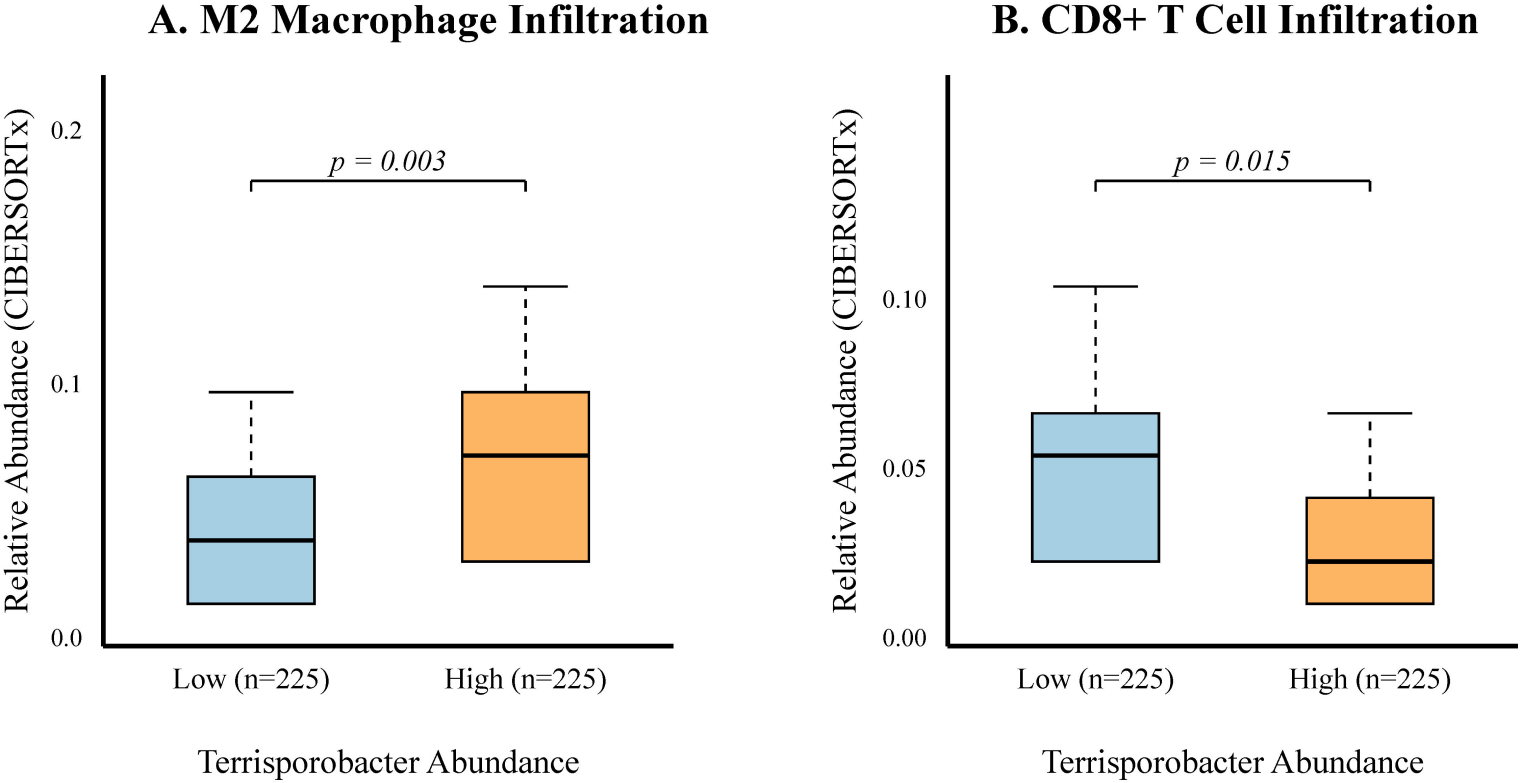
Association between intratumoral *Terrisporobacter* abundance and the tumor immune microenvironment in the TCGA-THCA cohort (n=450). Patients were stratified into high and low abundance groups by the median. Box plots compare the relative abundance of **(A)** M2 Macrophages and **(B)** CD8+ T cells between the two groups. X-axis represents *Terrisporobacter* Abundance group (Low vs. High); Y-axis represents Relative Abundance (CIBERSORTx) of immune cells. P-values were calculated using the Wilcoxon rank-sum test.

To explore the potential mechanism linking *Terrisporobacter* to poor prognosis, we analyzed the tumor immune microenvironment. We found that higher reads of *Terrisporobacter* mapped from tumor RNA-seq data were significantly correlated with an increased infiltration of pro-tumorigenic M2 macrophages (Spearman’s ρ = 0.25, p < 0.001) and a decreased infiltration of anti-tumor CD8+ T cells (ρ = -0.19, p = 0.008). Patients in the high-*Terrisporobacter* group exhibited significantly elevated M2 macrophage levels (p = 0.003) and reduced CD8+ T cell levels (p = 0.015) compared to the low-abundance group (**Figure 8**). These findings suggest that *Terrisporobacter* may contribute to PTC progression by fostering an immunosuppressive tumor microenvironment.

### 3.6 *Terrisporobacter* promotes *NTRK1* expression and proliferation in PTC cells *in vitro*

**Figure 9:**
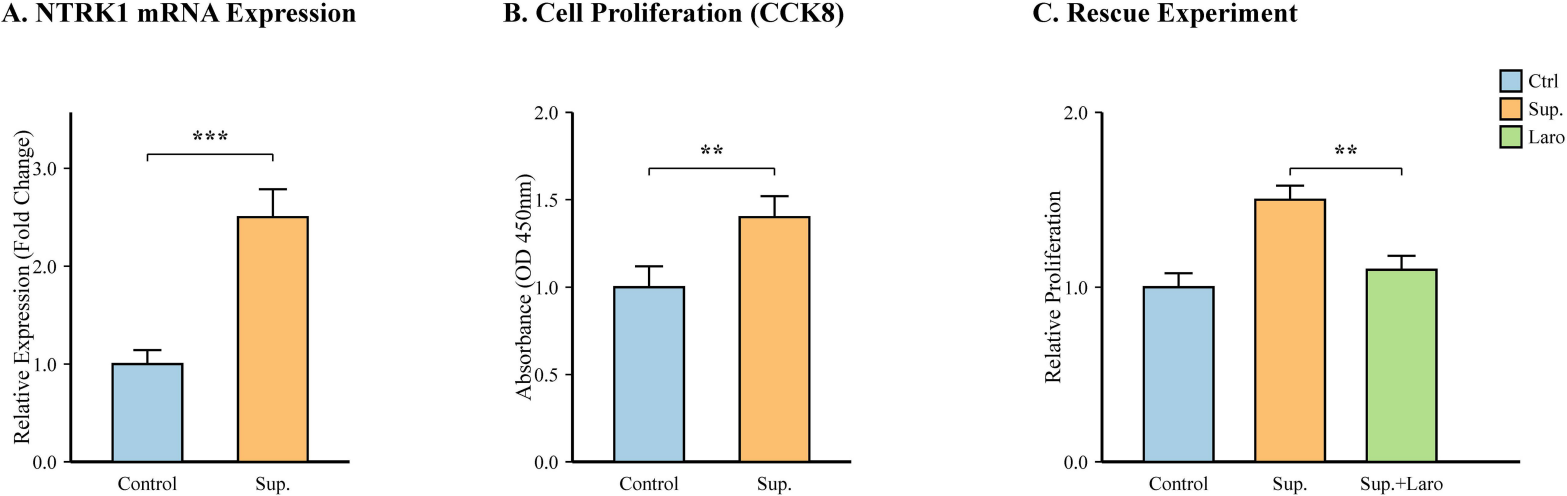
*In vitro* validation of the effect of *Terrisporobacter* on PTC cells. The PTC cell line TPC-1 was treated with sterile supernatant from *Terrisporobacter* culture (Sup.) or control medium. **(A)** Relative mRNA expression of *NTRK1* measured by qRT-PCR after 48 hours. **(B)** Cell proliferation measured by CCK8 assay after 72 hours. **(C)** Rescue experiment: TPC-1 cells were treated with control medium, bacterial supernatant, or supernatant plus the TRK inhibitor Larotrectinib (100 nM). Cell proliferation was measured by CCK8. The TRK inhibitor significantly reversed the supernatant-induced proliferation. Data are presented as mean ± SD from three independent experiments (n=3). ***p < 0.001, **p < 0.01 compared to control.

To directly test the causal link predicted by our MR and bioinformatic analyses, we performed *in vitro* experiments. After treating the PTC cell line TPC-1 with sterile supernatant from *Terrisporobacter* culture for 48 hours, qRT-PCR analysis revealed that the relative mRNA expression of *NTRK1* was significantly upregulated by approximately 2.5-fold compared to the control group (p < 0.001) (**Figure 9A**). Furthermore, a CCK8 assay demonstrated that the proliferation of TPC-1 cells was significantly enhanced after 72 hours of treatment with the bacterial supernatant (p < 0.01) (**Figure 9B**). To further confirm that the pro-tumorigenic effects were mediated by NTRK1, we performed a rescue experiment using the TRK inhibitor Larotrectinib. Treatment with Larotrectinib significantly attenuated the supernatant-induced cell proliferation (p < 0.01) (**Figure 9C**).

### 3.7 *Terrisporobacter* enhances invasion and induces de-differentiation in PTC cells

**Figure 10:**
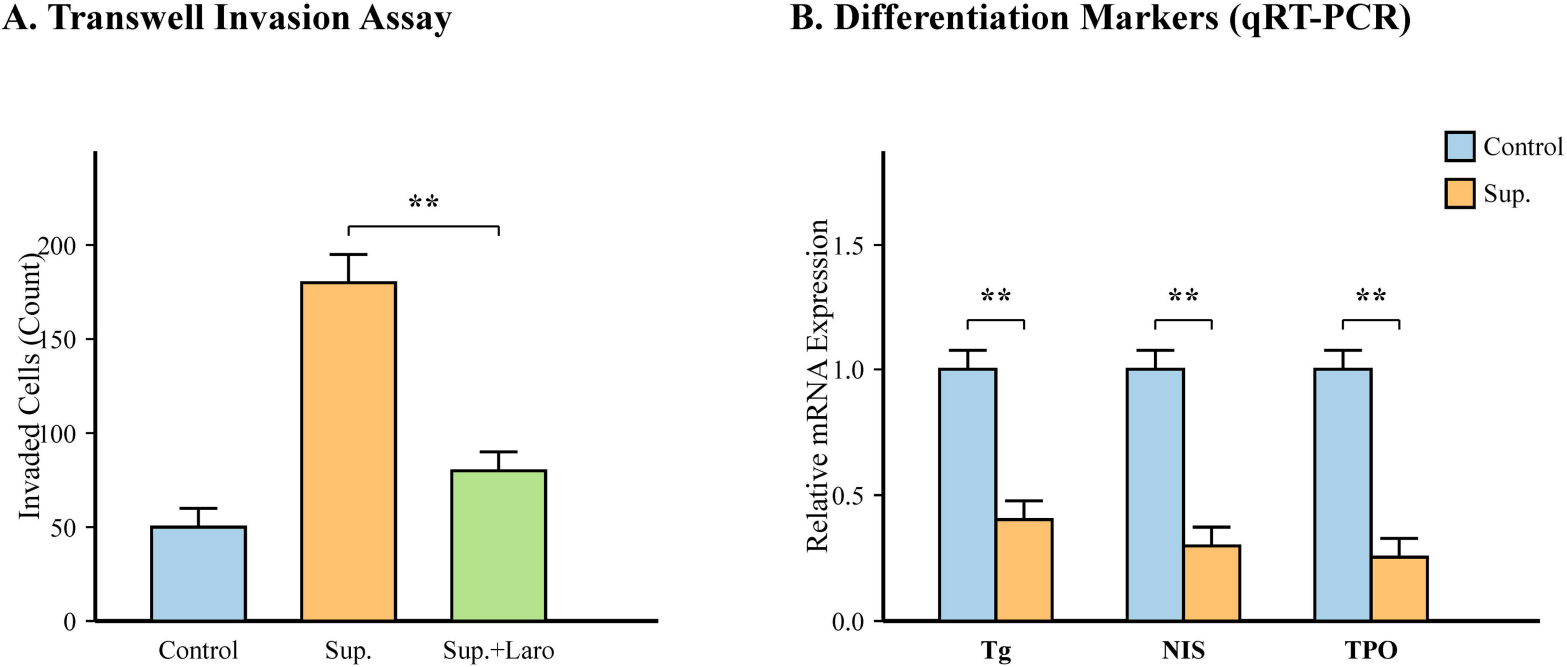
*Terrisporobacter* promotes invasion and de-differentiation in TPC-1 cells. **(A)** Quantification of Transwell invasion assay (n=3 independent experiments). The number of invaded cells was counted in three random fields per well. TPC-1 cells were treated with bacterial supernatant (Sup.) with or without Larotrectinib (Laro). Sup. increased invasion, which was reversed by Laro. **(B)** qRT-PCR analysis of thyroid differentiation markers Thyroglobulin (Tg), Sodium Iodide Symporter (NIS), and Thyroid Peroxidase (TPO) (n=3 independent experiments). Treatment with Sup. significantly downregulated all three markers. Data are presented as mean ± SD. **p < 0.01 vs Control.

Given that tumor progression involves invasion and de-differentiation, we assessed these phenotypes. Transwell assays showed that Terrisporobacter supernatant significantly increased the invasion of TPC-1 cells (p < 0.01), an effect that was also reversed by Larotrectinib (**Figure 10A**). Furthermore, qRT-PCR analysis revealed a significant downregulation of thyroid differentiation markers, including Thyroglobulin (Tg), Sodium Iodide Symporter (NIS), and Thyroid Peroxidase (TPO) (all p < 0.01), indicating that Terrisporobacter promotes a de-differentiated, more aggressive phenotype (**Figure 10B**).

### 3.8 Enrichment analysis of key genes

**Figure 11:**
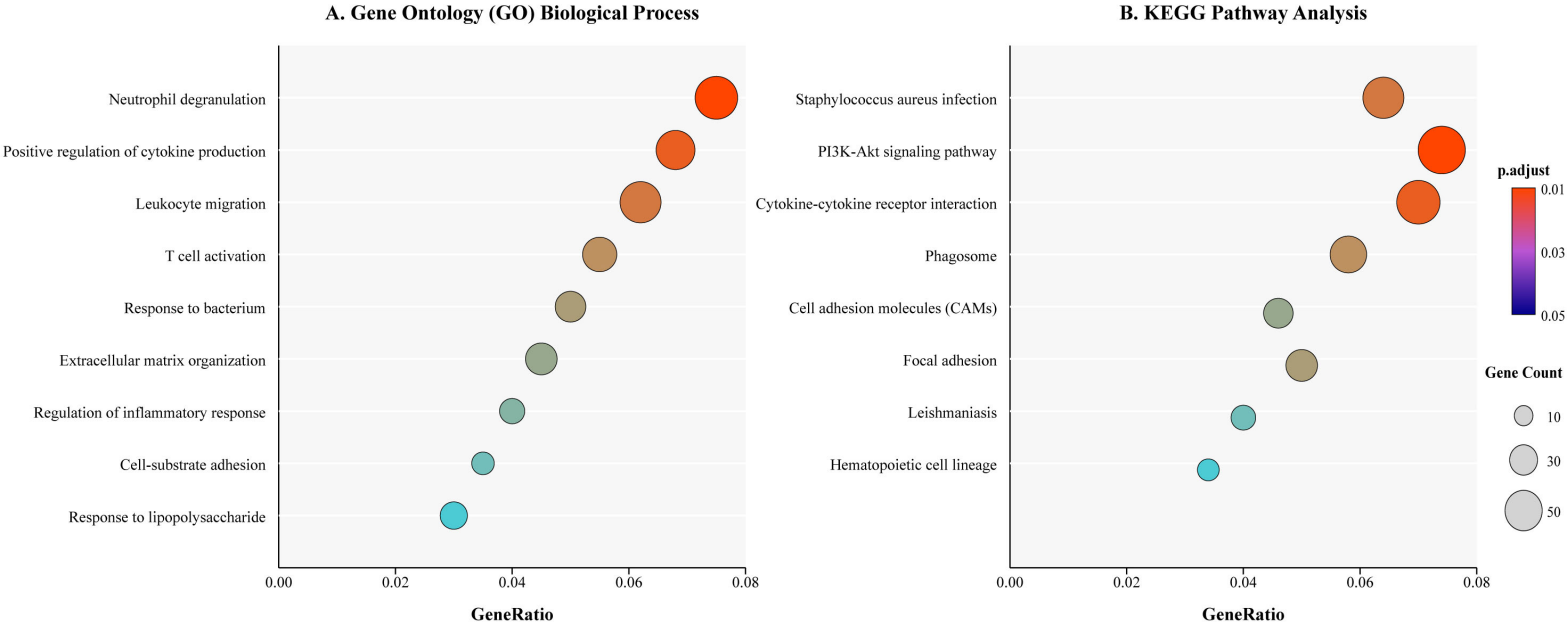
Enrichment analysis of genes related to the pathways in gut microbiota and PTC. **(A)** Gene Ontology (GO) enrichment results showing the top enriched Biological Process (BP) terms. **(B)** KEGG pathway enrichment analysis. Circle size is proportional to the number of genes enriched in a pathway, and color represents the adjusted p-value. (Genes identified from SNPnexus database mapping of microbiota-associated SNPs).

**Figure 12:**
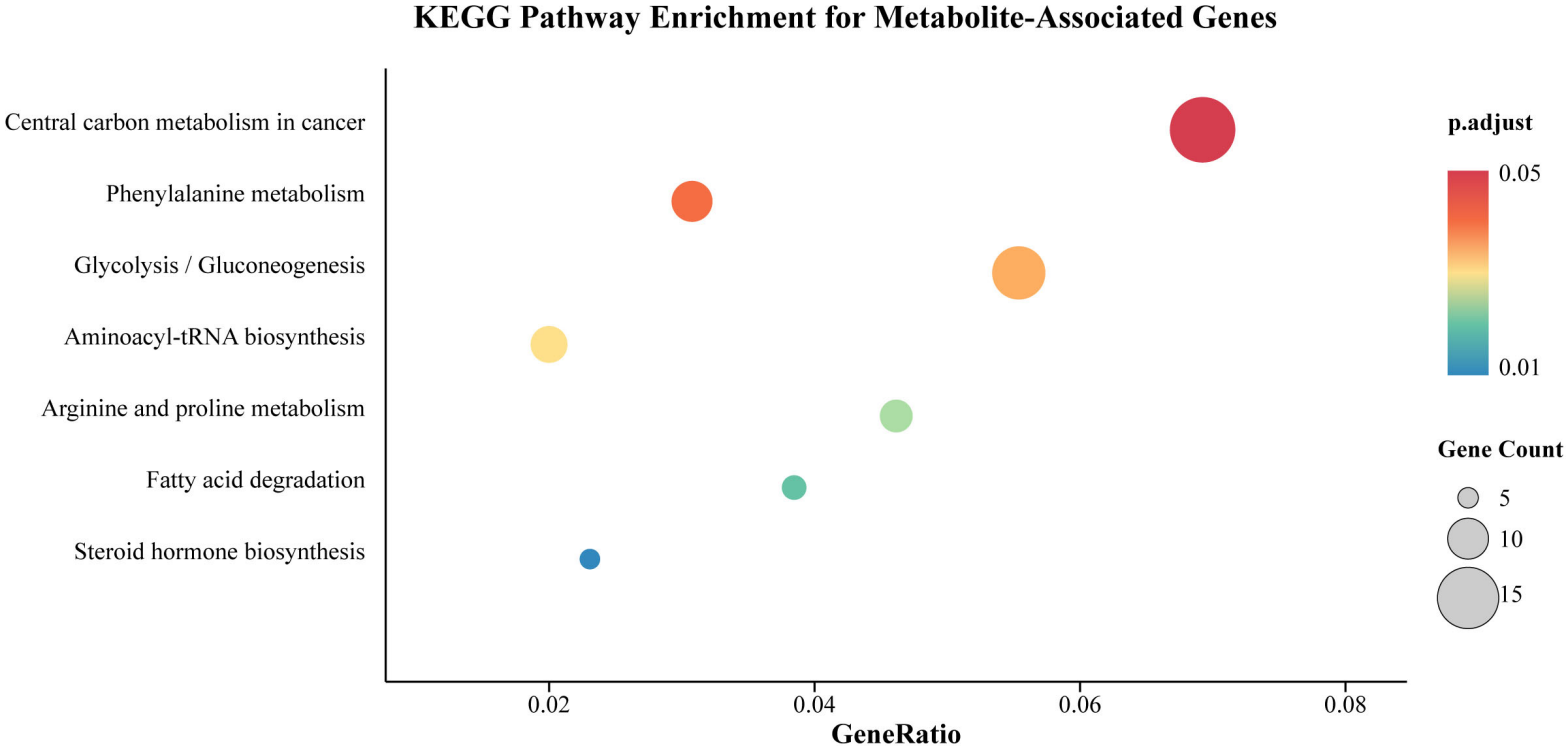
KEGG enrichment analysis of genes related to pathways in metabolites and PTC. Circle size is proportional to the number of genes enriched in a pathway, and color represents the adjusted p-value. (Genes identified from SNPnexus database mapping of metabolite-associated SNPs).

After threshold screening, the SNPs associated with the gut microbiota, metabolites, and papillary thyroid carcinoma were matched with genes in the SNPnexus database. For genes linked to gut microbiota, a total of 5 molecular functions (MF), 128 biological processes (BP) and 4 KEGG pathways were obtained (**Figure 11**), which were associated with the interaction between gut microbiota and papillary thyroid carcinoma. Additionally, for genes linked to metabolites, 7 KEGG pathways were identified (**Figure 12**), while no enrichment was observed in GO pathways.

Among the genes implicated in these pathways, several are known to be involved in tumorigenesis. The cAMP signaling pathway and central carbon metabolism in cancer pathways were identified as key pathways, with a total of 4 genes (such as GLS2, NTRK1, PDE4D and RYR2) involved, providing a mechanistic link between the exposures and PTC.

## 4 Discussion

The gut flora interacts with metabolites and/or the human immune system, making it a possible modulatory factor of PTC risk (22, 23). Over the last several decades, research on the microbiome and metabolomics has significantly improved our knowledge of the pathogenic process behind endocrine and digestive system tumors (24, 25). The majority of research on the functions of the metabolome and microbiome in disease came from case-control studies that tried to identify the changes that could be connected to certain illnesses. These kinds of research might point to correlations but cannot infer causality. MR is being used more and more by researchers to establish reliable causal links between risk variables and illness outcomes. This study represents a comprehensive effort to first establish causality using a robust two-sample MR design and then to validate these findings with clinical data, thereby strengthening the evidence for the role of the gut-thyroid axis in PTC. Crucially, by incorporating direct *in vitro* experimental validation, our study moves beyond computational prediction to provide tangible mechanistic evidence, solidifying the causal chain from a specific gut microbe to oncogene regulation and cellular behavior.

Our MR analysis identified a set of specific gut microbes causally associated with PTC risk. We revealed the possible correlations between Burkholderiales, Betaproteobacteria, Sutterella, and a reduced PTC risk, while Ruminococcaceae UCG014, Subdoligranulum, Methanobrevibacter and Terrisporobacter were associated with an increased risk. There have been reports that the possible negative relationship between Class Betaproteobacteria and other lipids in colorectal cancer patients (26). Unlike an early study, our findings underscore unfavorable connections between Genus Ruminococcaceae UCG014 and PTC. However, the abundance of thyroid cancer-enriched genera, including Ruminococcaceae UCG014 was negatively correlated with the levels of ApoB in a previous study (22). There was no report about relationship between Order Burkholderiales and PTC before, however, it is suggested that atrial fibrillation increased the abundance of Burkholderiales (27). A higher abundance of Genus Sutterella was found in complete remission group than that in non-remission group in patients with relapsed multiple myeloma, indicating Genus Sutterella may play a protective role in progression of cancers (28). Our finding of Sutterella’s protective effect aligns with this, suggesting a broader anti-tumor potential. In the preceding investigation, Family Ruminococcaceae was found to correlate with the development of hepatocellular carcinoma (29). The results we found were different from the protective effect of Family Ruminococcaceae, which was shown to be one of the high-risk factors for PTC in our results. In alignment with previous research, our findings shed light on notable contenders such as Methanobrevibacter and Terrisporobacter, which have surfaced as possible PTC risk factors (30). Nonetheless, there is still much to learn about the possible processes controlling the relationship between PTC and the gut flora. In this work, we have identified the genera that produce short-chain fatty acids (SCFAs), such as butyrate and propionate, such as Ruminococcaceae (31). The strong anti-inflammatory and anti-tumor effects of these SCFAs are well known (32). The discrepant roles of Ruminococcaceae across studies may reflect species-level differences and highlight the complexity of the microbiome’s influence.

A central finding of our work is the establishment of the pro-tumorigenic role of *Terrisporobacter* in PTC. Our multi-layered approach provides a cohesive narrative: the MR analysis first identified *Terrisporobacter* as a causal risk factor. This was then corroborated in the TCGA cohort, where its abundance was linked to advanced disease stage and, most importantly, to the expression of the oncogene *NTRK1*. The crowning piece of evidence came from our *in vitro* experiments, which demonstrated that soluble factors from *Terrisporobacter* directly upregulate *NTRK1* expression and promote PTC cell proliferation. Moreover, our supplementary experiments revealed that Terrisporobacter promotes tumor invasion and de-differentiation (downregulation of Tg, NIS, TPO), characteristics of aggressive thyroid cancers. The fact that the TRK inhibitor Larotrectinib could reverse these phenotypes strongly suggests that the NTRK1 pathway is the primary mediator of these effects. This confirms the biological plausibility of the *Terrisporobacter*-*NTRK1* axis and establishes it as a valid molecular mechanism in thyroid carcinogenesis.

Microbe-derived metabolites play a critical role in host–microbe interactions. Our study integrates this aspect by bridging the findings from our microbiome and metabolome MR analyses. In the current research, 4-androsten 3beta, 17beta diol disulfate 1, Myristoleate 14:1n5, Glucose, C-glycosyltryptophan and Ornithine were protective factors against PTC. In contrast, our study also identified metabolites associated with an increased risk of PTC, including Isovalerylcarnitine, Malate, Aspartate, and notably, Phenylalanine. Interestingly, some species of *Terrisporobacter* are known to be involved in amino acid metabolism, including the production of phenylalanine-derived compounds. This raises the compelling hypothesis that *Terrisporobacter* may exert its pro-tumorigenic effects, at least in part, by producing risk-associated metabolites like phenylalanine. While our study utilized bacterial supernatant to demonstrate a direct effect, a comprehensive global metabolomics or proteomics analysis of the Terrisporobacter culture supernatant is desirable in future studies to pinpoint the specific bioactive molecules—potentially phenylalanine derivatives or specific short-chain fatty acids—that drive the NTRK1 upregulation. This proposed “microbe-metabolite-gene” axis provides a comprehensive mechanistic framework that elegantly connects our disparate findings and warrants further investigation. Many of these metabolites, such as malate and aspartate, are central to cellular metabolism, and their dysregulation could fuel the metabolic reprogramming characteristic of cancer cells, an observation known as the Warburg effect (33).

A major strength of our study is the clinical validation of our MR findings in the TCGA cohort. This translational approach moves beyond mere statistical association to explore clinical relevance. We demonstrated that Terrisporobacter, a genus identified as a PTC risk factor by MR, was not only more abundant in advanced-stage tumors but also positively correlated with the expression of the oncogene NTRK1 and was linked to poorer patient survival. This provides a tangible biological link: Terrisporobacter may contribute to PTC aggressiveness by promoting an oncogenic signaling environment. Furthermore, our novel finding that *Terrisporobacter* abundance is associated with an immunosuppressive tumor microenvironment—characterized by increased M2 macrophages and decreased cytotoxic CD8+ T cells—adds another layer to its pro-tumorigenic mechanism and helps explain its link to adverse clinical outcomes. While we focused our immune microenvironment analysis on the microbial exposure itself to establish the gut-immune connection, future studies could further explore how downstream targets like GLS2 independently shape the TME. The cAMP signaling pathway and central carbon metabolism in cancer pathways were identified as key pathways, with a total of 4 genes (such as GLS2, NTRK1, PDE4D and RYR2) involved. Our clinical data strongly support the role of NTRK1 and GLS2. Glutaminase 2 (GLS2) is involved in glutamine metabolism and has been associated with thyroid cancer progression. Research has shown that GLS2 expression is lower in thyroid cancer tissues than in thyroid normal tissues, which may indicate a tumor-suppressive function (34, 35). Our findings that high GLS2 expression is linked to better survival supports its role as a tumor suppressor in PTC. Neurotrophic Receptor Tyrosine Kinase 1(NTRK1) is a receptor tyrosine kinase that participates in cell growth, differentiation, and survival (36). Aberrant activation of NTRK1 signaling has been implicated in thyroid cancer development. NTRK fusions are established therapeutic targets in thyroid cancer (37), and our data suggest that even without fusions, elevated NTRK1 expression, potentially influenced by the microbiome, is a significant driver of poor prognosis (38). Phosphodiesterase 4D (PDE4D) is an enzyme involved in the regulation of intracellular cyclic adenosine monophosphate (cAMP) levels (39). Ryanodine Receptor 2 (RYR2) is a calcium release channel whose dysregulation may contribute to thyroid cancer pathogenesis by affecting cell proliferation, migration, and apoptosis (40, 41).

This study has several limitations. First, the GWAS data were primarily from individuals of European ancestry, which may limit the generalizability of our findings to other populations. Second, while MR can mitigate confounding, it cannot completely eliminate the possibility of horizontal pleiotropy, although our sensitivity analyses showed no evidence of it. Third, the analysis of microbial abundance from TCGA RNA-seq data is an estimation and may not perfectly reflect the gut microbial composition; however, it represents a state-of-the-art method for leveraging large-scale cancer genomics datasets for microbiome research. Furthermore, the TCGA cohort has a marked female predominance (72.9%). While our sex-stratified analysis indicated that the correlation between Terrisporobacter and NTRK1 is present in both sexes, the prognostic impact was more statistically evident in females. This observation aligns with known sexual dimorphism in thyroid cancer immunity but necessitates larger male cohorts for validation in the future. Finally, our *in vitro* experiments, while providing crucial mechanistic support, utilized bacterial supernatant to simulate the effect of metabolic products and would benefit from future studies identifying the specific active compound(s).

## 5 Conclusion

**Figure 13:**
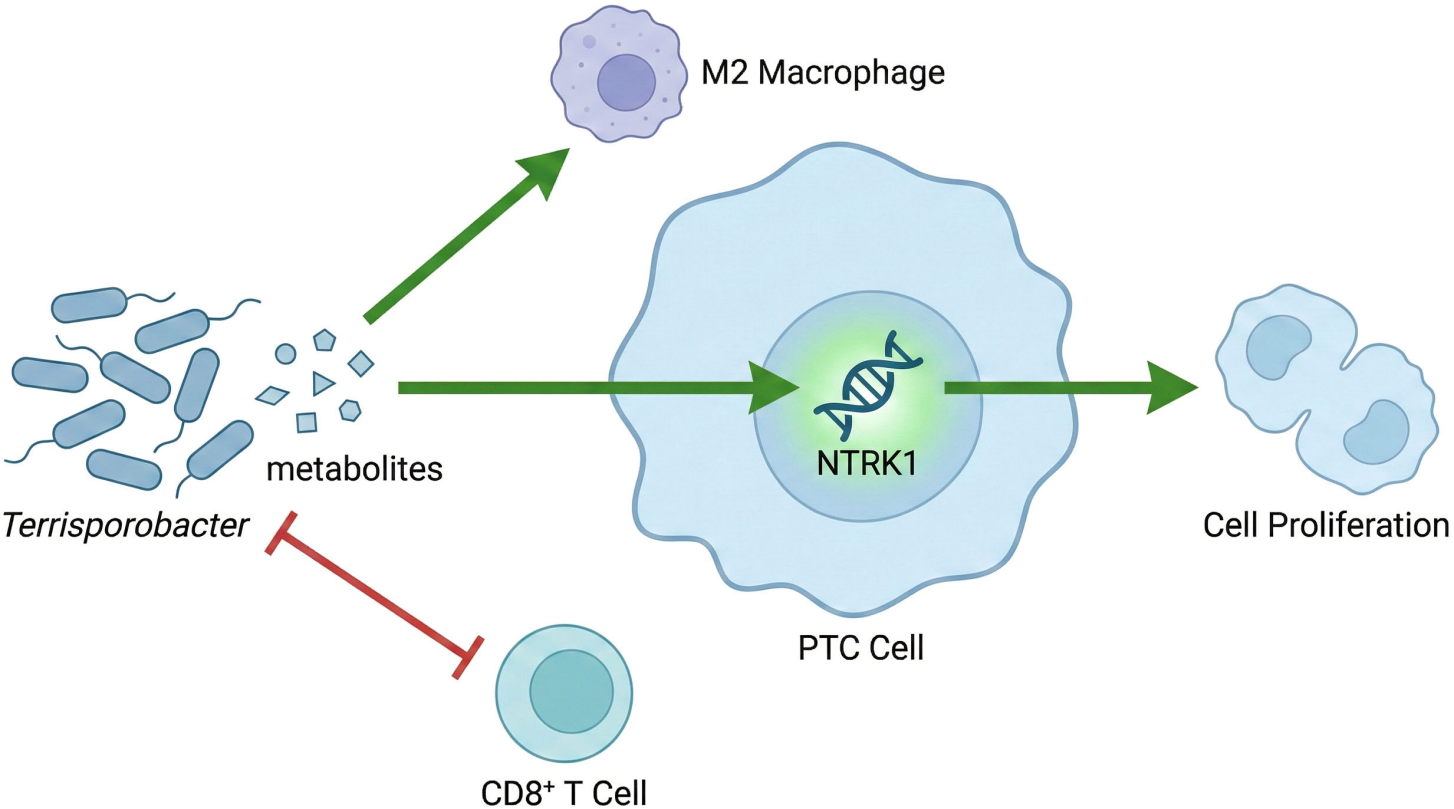
Proposed mechanistic model of the Terrisporobacter-NTRK1 axis in PTC progression. Dysbiosis of the gut microbiota leads to an increased abundance of *Terrisporobacter*. This bacterium promotes Papillary Thyroid Carcinoma (PTC) through two key pathways: (1) secretion of metabolites that directly upregulate the oncogene *NTRK1* in PTC cells, enhancing cell proliferation; and (2) remodeling of the tumor immune microenvironment by recruiting M2 macrophages and suppressing CD8+ T cells. Green arrows indicate promotion/activation; red T-bars indicate inhibition.

In summary, our results from a comprehensive, multi-layered investigation substantiate the proposition that specific alterations in gut microbiota composition and metabolite levels are causally associated with papillary thyroid carcinoma (PTC) risk. We provide novel and experimentally validated evidence that the gut microbe *Terrisporobacter* causally promotes PTC progression. Our work defines a new molecular axis where *Terrisporobacter*, potentially through its metabolic products, fosters an immunosuppressive tumor microenvironment and upregulates the oncogene *NTRK1*, leading to enhanced cell proliferation, invasion, and de-differentiation (**Figure 13**). These findings underscore that individual susceptibility to PTC may be influenced by the gut-thyroid axis and pinpoint the *Terrisporobacter-NTRK1* axis as a potential target for future prevention strategies and therapeutic interventions.

## Data Availability

The raw data supporting the conclusions of this article will be made available by the authors, without undue reservation.
