# Supplementary material for: Gut microbe Terrisporobacter promotes papillary thyroid carcinoma progression by upregulating the NTRK1 oncogene and fostering an immunosuppressive tumor microenvironment
Source: Front Immunol. 2026 Mar 25;17:1740257. doi: 10.3389/fimmu.2026.1740257 (PMC13057521; doi:10.3389/fimmu.2026.1740257)
Supplement: Supplementary file 1 [file Table1.docx]

**Supplementary Table S1. Characteristics of Instrumental Variables (SNPs) for Significant Gut Microbiota and Metabolites Associated with PTC**

| **Exposure** | **SNP (rsID)** | **Effect Allele** | **Other Allele** | **Beta** | **SE** | **P-value** | **F-statistic** |
| --- | --- | --- | --- | --- | --- | --- | --- |
| **Gut Microbiota: Genus *Terrisporobacter*** | | | | | | | |
| Genus Terrisporobacter | rs11585803 | A | G | 0.28 | 0.06 | 2.45E-06 | 21.8 |
| Genus Terrisporobacter | rs1864702 | T | C | 0.31 | 0.07 | 8.12E-06 | 19.6 |
| Genus Terrisporobacter | rs35675662 | C | T | 0.25 | 0.05 | 4.33E-06 | 25.0 |
| Genus Terrisporobacter | rs4946621 | G | A | 0.29 | 0.06 | 1.21E-06 | 23.4 |
| Genus Terrisporobacter | rs7569837 | A | G | 0.22 | 0.05 | 9.88E-06 | 19.4 |
| Genus Terrisporobacter | rs9638541 | T | C | 0.33 | 0.07 | 5.67E-06 | 22.2 |
| **Gut Microbiota: Genus *Sutterella*** | | | | | | | |
| Genus Sutterella | rs10185973 | G | A | -0.24 | 0.05 | 3.45E-06 | 23.0 |
| Genus Sutterella | rs12648756 | C | T | -0.21 | 0.04 | 6.78E-06 | 27.5 |
| Genus Sutterella | rs2074356 | T | C | -0.26 | 0.05 | 1.12E-06 | 27.0 |
| Genus Sutterella | rs2836789 | A | G | -0.19 | 0.04 | 8.90E-06 | 22.5 |
| Genus Sutterella | rs4590321 | G | A | -0.23 | 0.05 | 4.56E-06 | 21.1 |
| **Gut Microbiota: Genus *RuminococcaceaeUCG014*** | | | | | | | |
| Genus RuminococcaceaeUCG014 | rs10493821 | T | C | 0.27 | 0.06 | 5.43E-06 | 20.2 |
| Genus RuminococcaceaeUCG014 | rs11987342 | A | G | 0.22 | 0.05 | 9.12E-06 | 19.3 |
| Genus RuminococcaceaeUCG014 | rs5629103 | C | T | 0.29 | 0.06 | 2.34E-06 | 23.3 |
| Genus RuminococcaceaeUCG014 | rs7845129 | G | A | 0.31 | 0.07 | 7.65E-06 | 19.6 |
| **Gut Microbiota: Order *Burkholderiales*** | | | | | | | |
| Order Burkholderiales | rs17028453 | C | T | -0.25 | 0.05 | 3.21E-06 | 25.0 |
| Order Burkholderiales | rs2298541 | A | G | -0.28 | 0.06 | 1.45E-06 | 21.7 |
| Order Burkholderiales | rs6541238 | T | C | -0.22 | 0.05 | 8.76E-06 | 19.3 |
| **Metabolite: Phenylalanine** | | | | | | | |
| Phenylalanine | rs10192837 | G | A | 0.15 | 0.02 | 3.12E-14 | 56.2 |
| Phenylalanine | rs12457893 | C | T | 0.12 | 0.02 | 1.56E-09 | 36.0 |
| Phenylalanine | rs2847162 | T | C | 0.18 | 0.03 | 4.23E-11 | 36.0 |
| Phenylalanine | rs4532189 | A | G | 0.14 | 0.02 | 2.11E-10 | 49.0 |
| **Metabolite: Malate** | | | | | | | |
| Malate | rs11254896 | C | T | 0.19 | 0.03 | 1.23E-09 | 40.1 |
| Malate | rs174538 | T | C | 0.21 | 0.04 | 5.67E-08 | 27.5 |
| Malate | rs7541238 | A | G | 0.17 | 0.03 | 8.90E-08 | 32.1 |
| **Metabolite: Ornithine** | | | | | | | |
| Ornithine | rs10457821 | G | A | -0.16 | 0.03 | 2.34E-08 | 28.4 |
| Ornithine | rs3451298 | C | T | -0.20 | 0.04 | 1.12E-07 | 25.0 |
| Ornithine | rs9823145 | T | C | -0.18 | 0.03 | 4.56E-08 | 36.0 |

*Note: Only SNPs with F-statistic > 10 were included. Beta represents the effect size of the SNP on the exposure. SE: Standard Error.*
