# Supplementary material for: Gut microbe Terrisporobacter promotes papillary thyroid carcinoma progression by upregulating the NTRK1 oncogene and fostering an immunosuppressive tumor microenvironment
Source: Front Immunol. 2026 Mar 25;17:1740257. doi: 10.3389/fimmu.2026.1740257 (PMC13057521; doi:10.3389/fimmu.2026.1740257)
Supplement: Supplementary file 2 [file Table2.docx]

**Supplementary Table S2. Sex-stratified Analysis of Clinical Associations in the TCGA-THCA Cohort**

| **Analysis** | **Metric** | **Female (n=328)** | **Male (n=122)** |
| --- | --- | --- | --- |
| **Correlation: Terrisporobacter vs. NTRK1** | Spearman's ρ | 0.36 | 0.31 |
|  | P-value | < 0.001 | 0.002 |
| **Survival Analysis (High vs. Low NTRK1)** | Log-rank P-value | 0.005 | 0.08 |
|  | Hazard Ratio (95% CI) | 2.45 (1.31-4.58) | 1.85 (0.92-3.71) |
